# P-1685. Knowledge, Attitudes, and Practice of Healthcare Providers Regarding Oral Antibiotic Use in a Health System

**DOI:** 10.1093/ofid/ofae631.1851

**Published:** 2025-01-29

**Authors:** Emily Gammill, Emily Shephard, Hita Bhagat, Dominic Chan

**Affiliations:** Trinity Health, Portland, Oregon; Legacy Health, Portland, Oregon; Legacy Health, Portland, Oregon; Legacy Health, Portland, Oregon

## Abstract

**Background:**

Treating infections with oral (PO) antibiotics is associated with reduced risk for catheter associated infections, costs, and length of stay. However, barriers in appropriate transition to PO antibiotics exist.

Intravenous versus oral antibiotics by treatment indication

**Figure:**
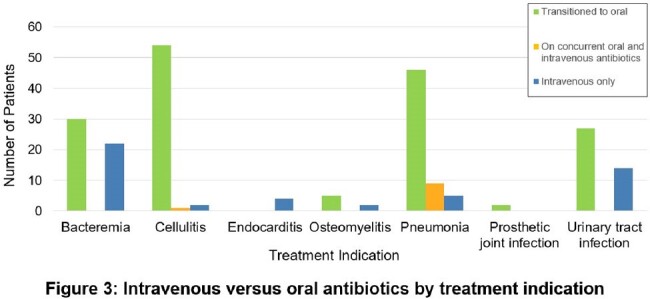

The most common treatment indications were pneumonia, cellulitis, bacteremia, and urinary tract infection. A lower proportion of patients with cystitis and bacteremia were transitioned to oral antibiotics compared to other indications.

**Methods:**

This study aims to understand the knowledge, attitudes, and practice of healthcare providers regarding PO antibiotics. This is a cross-sectional, descriptive survey followed by a multi-center, retrospective chart review. A web-based questionnaire was distributed electronically and promoted during meetings to healthcare providers. The retrospective chart review included admitted adult patients who were administered antibiotics for targeted infections. Patients that were pregnant, incarcerated, had inadequate PO absorption, or lack of source control were excluded. The primary outcome was to identify areas for antimicrobial stewardship intervention. This was done by identifying discordances between provider knowledge or attitudes and prescribing practices. Descriptive statistics and regression analyses were conducted.

**Results:**

The survey collected 119 total responses from pharmacists (47.1%), physicians (36.1%), medical residents (5.9%), and advanced practice providers (5%). Providers were most comfortable using PO antibiotics for cellulitis (88.2%), cystitis (86.6%), and community acquired pneumonia (82.4%), and were least comfortable using PO antibiotics for osteomyelitis (26.1%), prosthetic joint infection (16.8%), and endocarditis (14.3%). The most common barrier was the concern that PO antibiotics are not as effective (86.6%). The retrospective chart review included 224 patients, of which 164 were transitioned to PO antibiotics at any point. A lower proportion of patients with cystitis (60%) and bacteremia (57.7%) were transitioned to PO. The largest discordance between comfort using PO antibiotics and actual prescribing practices occurred with cystitis. Average duration of intravenous (IV) antibiotics were 4.2 days for pneumonia, 4.2 days for cellulitis, and 3.3 days for cystitis.

**Conclusion:**

Antimicrobial stewardship initiatives can be targeted at education on oral antibiotic options that have high efficacy, improving IV to PO conversion rates for cystitis, and implementing criteria for early transition to PO antibiotics.

**Disclosures:**

**All Authors**: No reported disclosures

